# Adjuvant Therapy for Stage IB Germ Cell Tumors: One versus Two Cycles of BEP

**DOI:** 10.1155/2018/8781698

**Published:** 2018-04-02

**Authors:** Robert A. Huddart, Alison M. Reid

**Affiliations:** Institute of Cancer Research and Royal Marsden Hospital FT, Sutton, Surrey, UK

## Abstract

Testicular germ cell tumours are the commonest tumours of young men and are broadly managed either as pure seminomas or as ‘nonseminomas'. The management of Stage 1 nonseminomatous germ cell tumours (NSGCTs), beyond surgical removal of the primary tumour at orchidectomy, is somewhat controversial. Cancer-specific survival rates in these patients are in the order of 99% regardless of whether surveillance, retroperitoneal lymph node dissection, or adjuvant chemotherapy is employed. However, the toxicities of these treatment modalities differ. Undertreating those destined to relapse exposes them to the potentially significant toxicities of 3-4 cycles of bleomycin, etoposide, and cisplatin (BEP) chemotherapy. Conversely, giving adjuvant chemotherapy to all patients following orchidectomy results in overtreatment of a significant proportion. Therefore, the challenge lies in delineating the patient population who require adjuvant chemotherapy and in determining how much chemotherapy to give to adequately reduce relapse risk. This chapter reviews the factors to be considered when adopting a risk-adapted strategy for giving adjuvant chemotherapy in Stage 1B NSGCT sand discusses the data regarding the number of BEP cycles to administer.

## 1. Background

Testicular germ cell tumours (TGCTs) are the commonest tumours of young men aged between 20 and 35 years. They are divided into those of pure seminoma subtype (that histologically resemble primordial germ cells) and those with elements that resemble extra embryonic tissue (with or without seminoma components), generally termed “nonseminomas.” Nonseminomatous germ cell tumours (NSGCT) make up roughly 50% of cases and are common in a younger age group than seminomas (median age around 25 years). A characteristic of TGCT is their sensitivity to cisplatin-based chemotherapy which means that, in contrast to most solid tumours, patients with metastasis are usually cured. The most commonly used schedule is “BEP,” consisting of bleomycin, etoposide, and cisplatin. This is highly successful chemotherapy and will cure around 85–90% of patients with metastatic disease and over 95% of those with good prognostic features (i.e., absence of visceral metastasis and low tumour markers) [1]. The cost of this is the risk of significant toxicity, both acute and late (e.g., renal damage, peripheral neuropathy, hearing damage, increased risk of cardiovascular disease, and second cancer; summarised in Table 1). Management of these patients is supported by monitoring tumour markers such as alpha fetoprotein (AFP) and human chorionic gonadotrophin (HCG), which are elevated in patients with metastatic NSGCT in around two-thirds of cases [2].

Around 75–80% of patients diagnosed with NSGCT present with no other clinical evidence of disease outside the testis on examination, imaging, or on tumour markers. These patients are termed Stage I and have excellent outcomes, with 5-year disease-specific survival over 98% [3]. Without additional treatment though, relapse occurs in around 30%.

Histopathological markers have been used to help predict the risk of recurrence. The best validated of these prognostic markers has been the presence or absence of lymphovascular invasion (LVI). Patients without LVI have around 15–20% risk of relapse, and if LVI is present, there is a 40–50% risk of recurrence [4]. This finding has been consistent across a number of large studies and is current standard of care in dividing patients into higher risk groups despite the modest predictive power; that is, even in low-risk group, 15–20% of patients will relapse and 50% of high-risk patients will remain in remission [1].

Other markers such as proportion of embryonal carcinoma (EC), rete testis invasion, and MIB-1 staining have been proposed, but results have not been fully validated. Tumours with no embryonal components generally have a low rate of recurrence [5–7]. Absence of undifferentiated embryonal carcinoma was one of the original MRC risk factors identified in the first MRC surveillance studies [6]. Some studies have suggested that the proportion of embryonal carcinoma may increase relapse risk [4, 8] though this does correlate with LVI and so has not been fully validated. MIB-1 has also been suggested to be associated with increased relapse risk, but in a recent study [8], an association could not be validated. Recently, CXCL12 expression has been proposed as a prognostic marker and validated in two data sets. On the basis of this marker and vascular invasion, three risk groups have been proposed including low (10% risk), intermediate (30–40% risk), and high (70%) risk groups though this remains to be validated.

A number of different approaches have been utilised in this patient group. In the past, removing the retroperitoneal lymph nodes has been practiced particularly in the United States and parts of Europe. Patients who are node negative (pN0) have a lower rate of subsequent recurrence (∼10–15%). Node-positive patients (pN1) may be cured by this approach but continue to have a significant rate of recurrence (∼30%). These patients have often been offered subsequent adjuvant chemotherapy (see below). Though undoubtedly some patients may avoid chemotherapy, this is at the cost of a major surgical procedure with the risk of attendant morbidity both immediate and long term. Patients still require follow-up as there remains a significant risk of relapse even in the pN0 group, and even without the use of adjuvant chemotherapy, a substantial number of patients will still be exposed to chemotherapy.

An alternative approach, based on pioneering work of Peckham and colleagues and subsequent MRC trials is to watch patients closely a policy termed active surveillance. This approach watches carefully with the aim of early relapse detection and treatment of recurrence by BEP chemotherapy. This approach has the virtue of avoiding treatment (and its attendant toxicities) except when necessary. The use of surveillance has grown over the years but is not necessarily trouble free. Patients who relapse require the use of full-dose chemotherapy [3]. The surveillance phase can cause psychological stress and can make it difficult for some patients to return to a normal lifestyle for fear of recurrence. Finally, there remains a concern regarding compliance. Several studies have shown a degree of noncompliance with follow-up in GCT patients. The fear is that noncompliers can relapse with more advanced disease with a poorer prognosis. This is a concern as a priori identification of noncompliance is difficult. For these reasons, the role of adjuvant chemotherapy has been explored especially in those at high risk of recurrence.

## 2. Adjuvant BEP Chemotherapy

An alternative approach to surveillance is to use adjuvant chemotherapy following treatment. This was first explored in the early 1990s after the success of using BEP chemotherapy in metastatic disease. It was argued that using more limited chemotherapy in those at high risk would restrict exposure to chemotherapy and prevent relapse. The key study of this approach was the MRC TE05 study. 114 patients were recruited with a predicted recurrence risk based on MRC risk factors of >50% [9]. Two patients recurred but one patient on histology review was proven to have a non-germ cell cancer, so in the 108 patients with centrally reviewed NSGCT, 1 relapsed and the 95% CI excluded a risk of relapse of 5%. This study was supported by similar smaller studies from Bern and Vienna [10, 11]. These data have been replicated in a number of reported studies totalling almost 1000 patients and a combined risk of relapse of <2% (summarised in Table 2). Most of the studies have used BEP for adjuvant treatment though a few studies have used variants of BEP. MD Anderson reported on substituting carboplatin for cisplatin [12], and the MRC replaced etoposide with vincristine [13], both achieving results similar to that achieved by BEP. The lower efficacy of carboplatin in metastatic disease and concerns regarding neurotoxicity, respectively, has meant that these approaches have not been adopted.

Following this work, adjuvant BEP has become an option for high-risk stage 1 NSGCT. Its use has been the subject of much debate. This is centered on the issue of late toxicity from BEP. The acute toxicities of BEP (Table 1) have been recognised for many years. Over the last decade, the risk of long-term effects have been better appreciated especially neuropathy, cardiovascular disease, and second malignancy. This has meant that opponents of adjuvant chemotherapy have advocated avoiding exposure of patients to the hazards of chemotherapy unless there is evidence of defined disease. On the other hand, proponents of adjuvant chemotherapy point to the lower doses used, and assuming this means less risk of toxicity. They also note the benefit of lower use of retroperitoneal lymph node surgery. Originally, the 2 cycles of BEP used adjuvantly was half of the dose of that used for metastatic disease. So, with a ∼50% relapse risk, the total number of cycles of chemotherapy needed to be delivered (“burden” of chemotherapy) was similar with either approach, though distributed differently in the population. However, with a MRC/EORTC randomised trial [12] showing that 3 cycles of BEP was sufficient for most patients, the balance in terms of burden of chemotherapy was in favour of a surveillance approach (i.e., assuming a 50% relapse risk); for every 100 adjuvant patients, 200 cycles of BEP would be delivered compared to 150 cycles for 100 surveillance patients undergoing 50 relapses. This has led to the exploration as to whether less adjuvant chemotherapy may be sufficient.

## 3. Single-Cycle Adjuvant BEP

At the Royal Marsden, we initially investigated a single cycle of BEP in patients with intermediate risk of recurrence. In 22 patients with approximately 20% risk of recurrence (i.e., 4-5 patients expected to recur), no active recurrences were noted though 1 patient required resection for teratoma differentiated [20]. In a similar higher risk group (defined as presence of vascular invasion or predominant embryonal carcinoma) in 42 patients, Westermann and colleagues reported a single active relapse compared to an expected rate of around 15 patients [21] (updated in [22]).

This approach has now been tested in 3 larger studies (Table 3). In the first of these, Albers and colleagues in the German germ cell cancer study group conducted a randomised trial comparing a single cycle of BEP versus RPLND [23]. In 191 patients randomised to BEP, 2 relapses were seen (1 of which was mature teratoma). In the RPLND arm, 32/173 patients were node positive and received adjuvant chemotherapy, and 15 node-negative patients relapsed (equating to 47 patients with active disease, 27%). The Scandinavian Swenoteca [24] group adopted a single cycle of BEP for intermediate- and high-risk patients as a population-based treatment protocol. In their latest report on 571 patients and a median follow-up of 7.9 years, 3.2% of patients with lymphovascular invasion (*n* = 258) and 1.6% patients with no lymphovascular invasion (*n* = 255) have relapsed with only one patient dying of multiply relapsed TGCT.

The latest study to report is the UK 111 study [25]. This large prospective nonrandomised study was designed to specifically exclude a relapse risk of over 5% after a single cycle of BEP. This study recruited 246 patients and reported after a median follow-up of 39.9 months and with a minimum follow-up of 2 years in 91% of patients. Four patients have had a malignant recurrence and 3 a TD recurrence. The primary end point was 2-year malignant recurrence rate which was 1.3% (95% CI 0.4–4.0%) and thus met its aim to exclude a 2-year recurrence rate of 5%. A full publication is awaited.

## 4. Current Perspectives

It is our opinion that the data discussed are now robust enough to conclude that 1 cycle of BEP can successfully reduce the risk of recurrence to less than 5%. The additional benefit of adding a second cycle in terms of risk reduction of relapse is small and, in our judgement, is not sufficient to justify the additional toxicity. Using a single cycle of BEP would reduce the overall use of chemotherapy in the “high-risk” vascular invasion population. Using the model described above, treating 100 patients with single-cycle adjuvant BEP would give 100 cycles to this population compared to 150 cycles if it is assumed that 50% relapse on surveillance. In addition to amount of chemotherapy, the balance of who receives is very different either spread across the whole population or concentrating, with greater exposure, in those with proven disease. There is no evidence in any of the studies of adjuvant chemotherapy or surveillance that use of either approach affects overall survival. This has prompted a vigorous debate between supporters [26–28] of adjuvant chemotherapy and surveillance as to the optimal approach, both in seminoma and nonseminoma. To our mind, to exclusively offer either approach, given lack of survival difference, is inappropriate. We attempt to present a balanced argument of the pros and cons of the two approaches stressing the impact on relapse and discussing what is known regarding immediate- and long-term toxicity (including risk of cardiovascular disease and second malignancy). The idea of adjuvant toxicity seems intrinsically attractive to many, with the wish to get treatment over with and avoid future uncertainty of relapse as common levers for deciding on adjuvant chemotherapy.

### 4.1. Future Approaches

Adjuvant chemotherapy has usually been used for patients found to be at higher risk of recurrence. The adoption of single-cycle adjuvant chemotherapy raises the question of whether patients with lower risk should be offered this treatment. The Swenoteca group allowed patients with lower risk of recurrence to receive adjuvant BEP in their protocols and reported a less than 2% relapse risk [24]. We do not offer this approach routinely as this would expose 80% or more of patients to BEP who would not have relapsed and increases the overall burden of chemotherapy. There may be exceptional instances such as when surveillance or full course of BEP would be difficult when it might be considered.

The landscape of adjuvant therapy could be changed if there was better prognostication. We recently reported a new prognostic index that could split stage 1 NSGCT patients into 3 broad groups based on vascular invasion and whether they had expression of CXCL12 or had near 100% embryonal content [8]. Using these criteria, we identified a small group (10–15%) with a very high recurrence risk (>70%) and a significant group (∼40–50%) with a low risk of recurrence (∼10%) with the remainder having a 30–40% recurrence risk. This index needs further validation but may have implications for decision making regarding adjuvant therapy. We believe most clinicians would agree the low-risk group should be surveyed and the high-risk group would benefit from adjuvant therapy. The intermediate group may be more controversial. The total burden of chemotherapy for surveillance and adjuvant chemotherapy would be roughly similar. These patients might reasonably be surveyed but many may consider the risk high enough to justify adjuvant chemotherapy if a single cycle of BEP is used.

This landscape is not static. More sophisticated genetic profiling may be on the horizon [29], and whilst data are emerging, that microRNA analysis may be a sensitive indicator of subclinical disease [2, 30]. Any impact on prognostication may affect the balance between surveillance and adjuvant therapy.

It is possible that the increasing confidence in the use of adjuvant BEP could change the treatment algorithm in other areas. For instance, could single-cycle BEP with less-invasive surgical techniques lead to a resurgence of surgical management of low-volume (stage 2) NSGCT [31]?

## 5. Conclusion

Single-cycle BEP is effective at reducing the risk of recurrence of stage 1 NSGCT to less than 5% and should be the preferred over two cycles of BEP if adjuvant therapy is chosen rather than surveillance. However, surveillance approaches can legitimately still be offered to all patients.

## Figures and Tables

**Table 1:** Toxicities of BEP chemotherapy.

Dose-related toxicities of BEP	Non dose related	Unknown relationship to dose
Infertility	Febrile neutropenia	Second malignancy
Peripheral neuropathy	Alopecia	Cardiovascular disease
Ototoxicity	Nausea/vomiting	
Raynaud's phenomena		
Fatigue		
Skin toxicity		
Avascular necrosis hip		
Pneumonitis/lung fibrosis		
Renal damage		
Anaemia		
Metabolic syndrome		

**Table 2:** Studies of 2 cycles of adjuvant chemotherapy in testicular nonseminomatous germ cell tumours.

Study	Institution	Chemotherapy	Eligibility	Median follow-up (months)	*N*	Number of malignant relapses (all relapses)
Cullen et al. [9]	MRC, UK	BEP	MRC >50% risk	NS	104	1 (1)
Pont et al. [11]	Vienna	BEP	VI	79 (range, 27 to 119)	40	1 (2)
Bohlen et al. [10]	Berne	BEP/PVB	≥pT2/EC	93 (range 32 to 146).	58	0 (1)
Germa-Lluch et al. [14]	Spanish germ cell group	BEP (90%)	≥pT2	NS	168	1
Chevreau et al. [15]	Toulouse	BEP	VI or EC	113.2 (range 63–189)	40	0
Oliver et al. [16]	Anglia, UK	BEP BOP	MRC >30% risk	NS	28 74	1 (1) 2 (2)
Amato et al. [12]	MD Anderson	CEB	^∗^MDA high risk	38	68	0 (1)
Guney et al. [17]	Istanbul	BEP	^∗^MDA high risk	26 (range 10–60)	71	3 (4)
Dearnaley et al. [13]	MRC, UK	BOP	VI	70	115	2 (2)
Bamias et al. [18]	Hellenic germ cell group	BEP	≥pT2/EC	79	142	1 (1)
Mezvrishvili and Managadze [19]	Tbilisi	BEP/EP	VI	NS	41	0 (1)
Total					949	12 (17) 1.3% (1.8%)

^∗^MDA high risk: pre-op AFP > 80 ng/ml, VI or >pT2. Chemotherapy drugs: B, bleomycin; C, carboplatin; E, etoposide; O, vincristine; P, cisplatin; V, vinblastine. MRC, Medical Research Council; VI, lymphovascular invasion present; EC, embryonal carcinoma.

**Table 3:** Summary of studies of single-cycle adjuvant BEP chemotherapy.

Study	Eligibility	Median follow-up (months)	*N*	Number of malignant relapses (all relapses)	Predicted relapse risk	Predicted number of relapses
Gilbert (RMH) [20]	1 or 2 MRC RF	120	22	0 (1)	20%	4
Westermann (Switzerland) [21]	VI+ >50% EC	99	13 29	1	30%	13
Albers (GCSCG) [23]	Any	56	191	1 (2)	26%	50
Tandstad [24] (SWENOTECA)	VI+ VI−	95	258 255	7 (8) 3 (4)	42% 12%	108 31
111 study [25]	VI+	40	236	4 (7)	42%	99
Total			**1004**	**16 (23)1.6% (2.3%)**		305^∗^

^∗^23/305 equals 7.5% of predicted relapses.
